# Anaphylaxis by inhalation of allergenic proteins from Anisakis simplex

**DOI:** 10.1186/2045-7022-3-S2-P13

**Published:** 2013-07-16

**Authors:** Dalhila Janett Garcia Navarro, Ana Montoro De Francisco, Tomas Chivato Perez, Francisco Javier Membrillo De Novales, Maria Angeles Nuñez Hernandez, Belen De Mateo Hernandez, Manuel Fernandez Lopez, Jose Maria Mateos Galvan

**Affiliations:** 1 Hospital Central de la Defensa “Gómez Ulla”, Madrid, Spain; 2 Hospital Central de la Defensa “Gómez Ulla'', Allergy Department, Madrid, Spain; 3 Hospital Central de la Defensa “Gomez Ulla”, Internal Medicine Department, Madrid, Spain

## Background

Anaphylaxis can affect 20% of patients sensitized to Anisakis simplex. There have been reports of occupational asthma due to inhalation of allergens in patients who handle raw fish. Anisakis has several proteins which are capable of producing sensitations, primarily by ingestion, but may also occur through contact or inhalation.

## Method

We report a 50 year old patient, who works as a cook. A year ago she presented two episodes of anaphylaxis after eating undercooked fresh fish. Thereafter she removed raw fish from her diet. Ten months later after handling fish (eviscerating it), an hour after hand injuring, she presented itching hands and head, epigastric pain, nausea and severe generalized urticaria. Attended the emergency room with adrenaline, corticosteroids, antihistamines, with good response. She manipulates fish daily with gloves because of her profession. Prick test (PT) with allergenic extracts of fish, Anisakis, food (Alk Abelló).Total and specific IgE to Anisakis, white and blue fish (Thermo Fisher). Gastroscopy.

## Results

Negative PT both raw and cooked whiting. Anisakis simplex PT positive both immediate and 48h later reading. Allergic response test against aeroallergens negative. Total IgE 1821KU / l. Specific IgE levels were always kept high Anisakis:> 100KU / l.Normal Gastroscopy: Anisakis larvae not observed. Not taking biopsy.

## Conclusion

We report a case of anaphylaxis following ingestion, contact and inhalation of various Anisakis simplex proteins in a patient previously sensitized by digestive tract. The positivity of tests carried out in vivo and in vitro against Anisakis with negativity against fish proteins, confirms the diagnosis of anaphylaxis by inhalation, because the patient had not returned to eating fish.

